# Supplementary material for: Optimization of In-Situ Exosome Enrichment Methodology On-a-Chip to Mimic Tumor Microenvironment Induces Cancer Stemness in Glioblastoma Tumor Model
Source: Cells. 2025 May 6;14(9):676. doi: 10.3390/cells14090676 (PMC12071966; doi:10.3390/cells14090676)
Supplement: Supplementary file 1 [file cells-14-00676-s001.zip › cells-3589563-supplementary.pdf]

cells

MDPI

Supplementary Materials

A. Study design

1. Estimating the local concentration of a chemical secreted from tumor cells within the TME

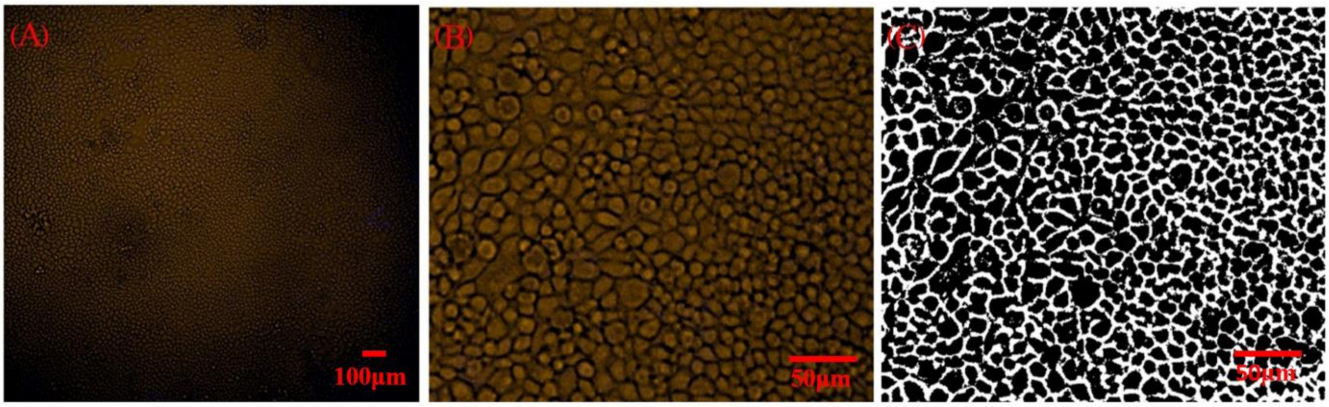

Figure S1. 2D culture of U87 cells. (A) a 100% confluent culture of U87 cells, red scale bar is 100µm (B) an enlarged crop of 2D confluent culture of U87 cell in a plate. (C) Binary mask of the image B to measure the interstitial space between the cells which works as TME in solid tumor. The total surface of interstitial space averages 29% of total surface of cell culture.

To estimate the volume of extracellular matrix that acts as TME in a tumor body, a 2D culture of U87 cells was conducted until it reached 100% confluency on the plate (Figure S1A). By creating a binary (black and white) mask model (Figure S1C) of the cells on the plate and using ImageJ software (ver. 1.54f), the ratio of the interstitial space between the cells to the total culture surface was measured, averaging 29%.

In parallel, 3D tumor organoids (tumoroids) were cultured using the hanging drop method in 20 µL of DMEM medium supplemented with 10% FBS, with about 3000 cells in each droplet. The tumoroids grew to about 300 µm in diameter. One hundred tumoroids were subcultured in 2 mL of medium (20 µL tumoroid $^{-1}$) for two days, and the concentration of TGFβ in the medium was about 20 ng mL^{-1} using ELISA.

Based on the calculated volume of each tumoroid and considering that a maximum of 29% of the tumoroid volume constitutes the extracellular matrix, the total amount of TGFβ distributed in 20 µL of culture medium was 0.4 ng, originating from one organoid. Therefore, in a solid tumor, this amount of growth factor is distributed in the volume of the interstitial space of the cells, which is a maximum of 29% of the total tumor volume. The concentration of TGFβ distributed in the volume of the 300 µm diameter tumoroid would be 306,513 ng mL^{-1}. Thus, the local concentrations of secreted chemicals are several folds higher than the measured concentrations by conventional methods.

2. Response Surface Methodology

Response surface methodology is a collection of mathematical and statistical techniques used to design experiments when dealing with multiple variables and responses. This study focuses on three vital responses: cell proliferation, glucose content, and exosome content, while limiting the variables to the time interval for replacing fresh culture medium and the volume of replaced culture medium.

2.1. Optimization of Cell Proliferation

Optimization of cell proliferation was first carried out by determining the pattern of data distribution (**Figure S2**). The plot shows that the dataset follows a normal distribution, indicating that the data is appropriate for statistical optimization.

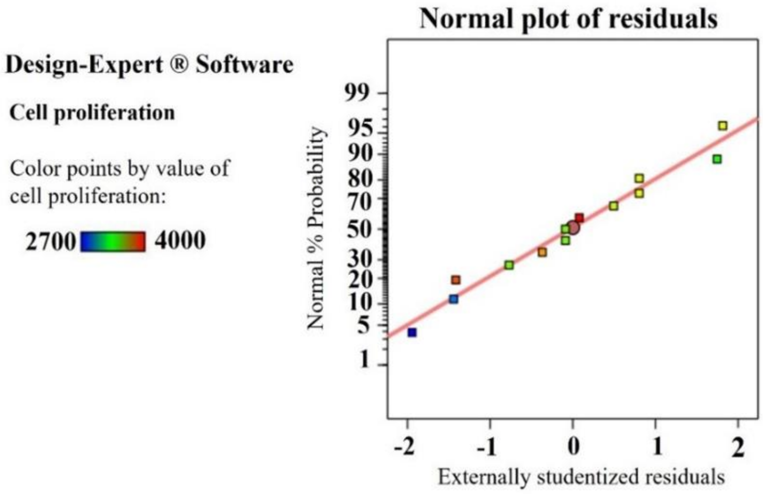

Figure S2. Normal plot of residual of cell proliferation data distribution.

Five models—mean, linear, 2FI, quadratic, and cubic—were tested. Among all, a linear model was chosen to analyze the data as it has the highest order polynomial with a significant sum of squares (1.354E+06), significant Lack-of-Fit, and highest R^2 (0.82). Analysis of the variance (ANOVA) for the response surface linear model was conducted to obtain the significance level of the fitted model and the factors affecting cell proliferation.

Table S1. Shows the ANOVA for the linear model.

Source	Sum of Squares	df	Mean Square	F-value	*P*-value	
Model	1.354E+06	2	6.770E+05	23.12	0.0002	significant
A-Time	4.587E+05	1	4.587E+05	15.66	0.0027	
B-Volume	8.954E+05	1	8.954E+05	30.57	0.0003	
Residual	2.928E+05	10	29283.85			
Lack of Fit	2.698E+05	6	44973.09	7.82	0.0333	significant
Pure Error	23000.00	4	5750.00			
Cor Total	1.647E+06	12				

The significance of each coefficient was indicated by the F and P-values, which also demonstrate the interaction strength between each independent variable. Values of 'Prob > F' less than 0.005 indicate that the

model is significant and vice versa. Based on Table S1, the p-value of the model was (P-values < 0.001) and the F-value was large (23.12), implying that the model was significant for interpreting the data. The "Lack of Fit P-value" was 0.0333, indicating that the Lack of Fit was significant relative to the pure error.

Because our goal was to increase exosome concentration rather than maintain proliferation, we were able to achieve controlled and optimal conditions in which cell differentiation was successful. Despite the use of such a difference in the normal growth model, the significance of the lack of fit for P-values of 0.033 is reasonably acceptable, completely logical, and natural. Based on the ANOVA analysis, two factors showed a significant impact on cell proliferation. The equation in terms of actual factors can be used to make predictions about the response for given levels of each factor. The coefficient estimate represents the expected change in response per unit change in factor value when all remaining factors are held constant. Thus, the experimental values obtained from the central composite design (CCD) were regressed using a linear equation, and the regression equation, expressed in terms of the actual factors, is shown below:

$$\text{Cell proliferation (Number of cells mm}^{-2}) = +\,3151.82753 - 67.72971(\text{Time}) + 3.78492\,(\text{Volume}) \quad \textbf{(1)}$$

The graph of observed values vs. expected values is shown in **Figure S3**, which aids in detecting observations that the model fails to predict. The 45° line should split the data points evenly, which shows that at the lower boundary of cell proliferation (about 2800), the model predicts higher cell proliferation than the real results. The deviation from normal cell proliferation behavior from normal linearity confirms that the imposed condition through enrichment of exosome content can induce changes in cell behavior, but to such an extreme that it causes cell death. This is discussed in the article results section.

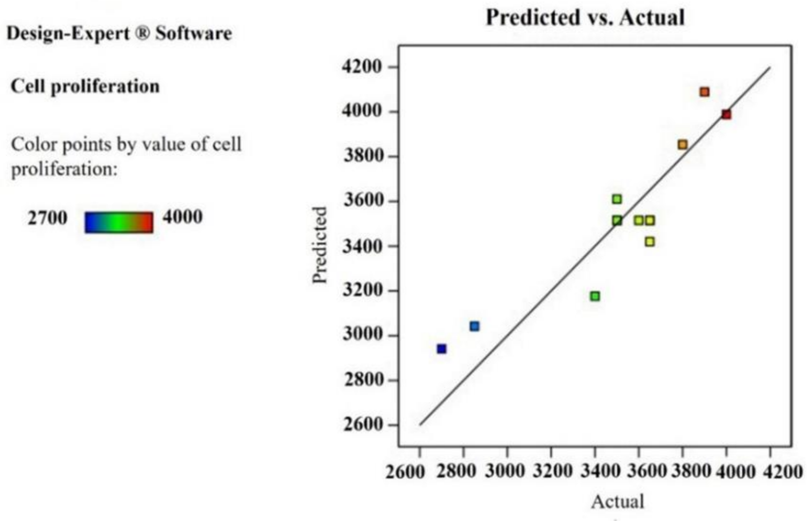

Figure S3. Correlation between the actual and predicted cell proliferation

2.1.2. Effects of the Variables on Cell Proliferation

The individual effects of time and volume showed a negative and positive impact on cell proliferation, respectively, and a one-factor plot for these two factors was generated (**Figure S4**). Based on this figure, it

showed that increasing the time interval for the replacement of fresh culture medium from 5 to 13.5 hours had a negative influence on glucose content.

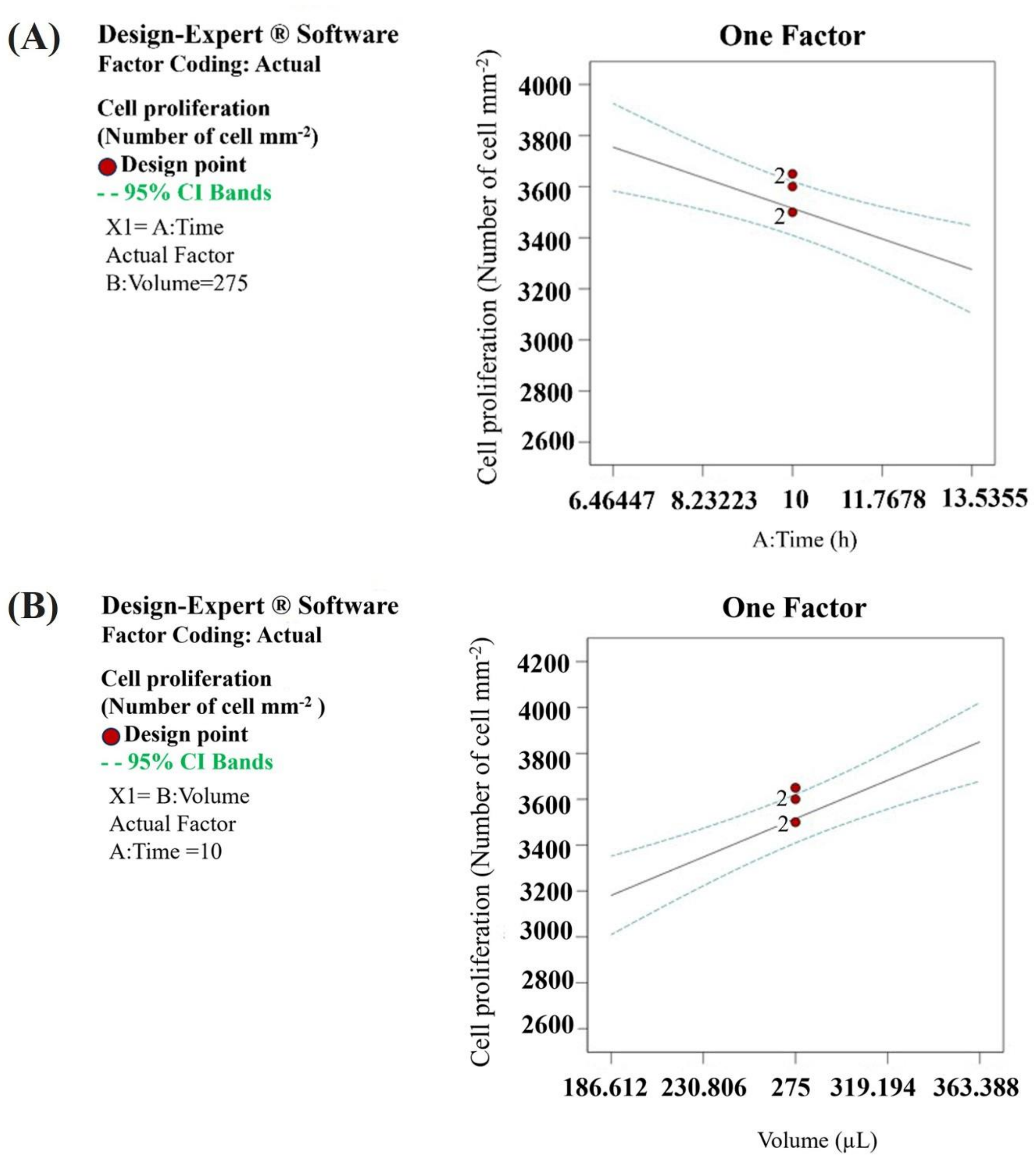

Figure S4. One factor plots of Time (A) and Volume (B) on Cell proliferation

The three-dimensional (3D) response surface was plotted to study the interaction of the two factors on cell proliferation. This type of graphical visualization allows the relationships between the experimental levels of each factor, the response, and the type of interactions between test variables, which is necessary to establish the optimal medium components and culture conditions. **Figure 2e** shows the 3D plot of the interaction between time (A) and volume (B) on cell proliferation.

The interaction of these two factors had a significant impact on cell proliferation with P-values < 0.005 (**Table S1**). Maximum cell proliferation occurs when the time interval for the replacement of fresh culture medium (A) and the volume of replaced culture medium (B) were at the center point and maximum levels, respectively (**Figure 4a**). This could be explained by the fact that glucose represents not only other nutrients but also some metabolites and oxygen, which are vital for the growth of cells. When these two factors are at insufficient levels, it consequently hurts cell proliferation.

2.2. Glucose Content Optimization

The maximum glucose content was in standard order 3 (99.05 mg dL^{-1}) and the lowest was in standard order 2 (36.7 mg dL^{-1}) (See **Table 1** in the method section). The normal plot of data distribution for glucose content is shown in **Figure S5**. The plot established that the dataset follows a normal distribution and is suitable for further optimization. Similar to the response of cell proliferation, the linear model was the fittest model for the regression of the experimental data. ANOVA for the linear model is shown in **Table S2**.

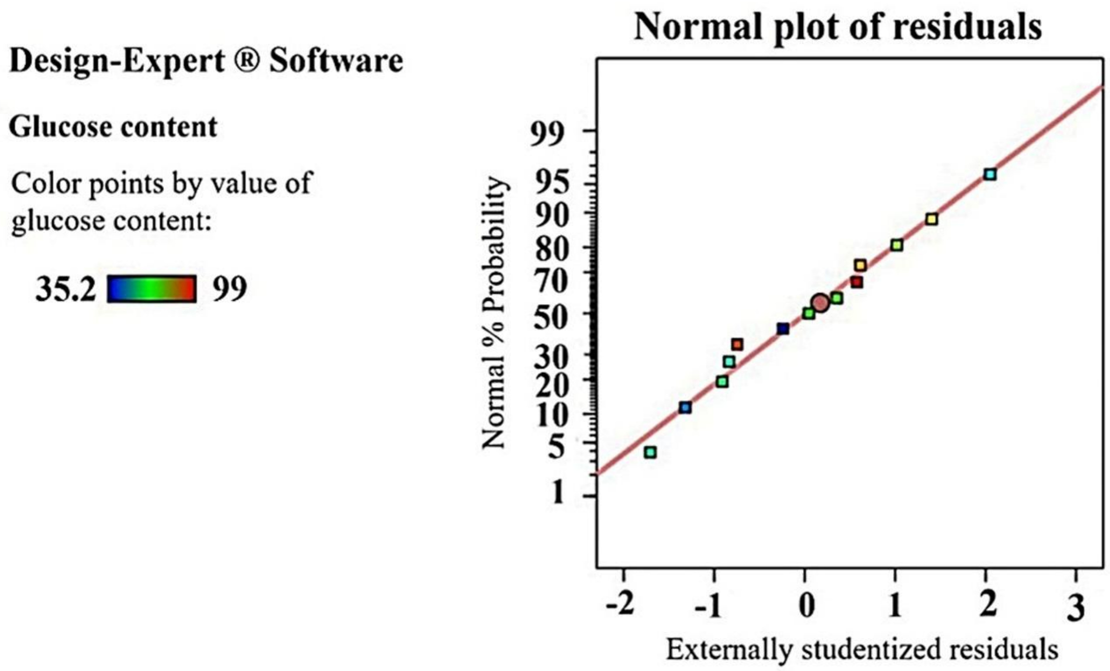

Figure S5. Normal plot of residual of glucose content data distribution.

Table S2. ANOVA for glucose content

Source	Sum of Squares	df	Mean Square	F-value	*P*-value	
Model	4141.21	2	2070.61	41.11	< 0.0001	significant
A-Time	3059.49	1	3059.49	60.74	< 0.0001	
B-Volume	1081.72	1	1081.72	21.48	0.0009	
Residual	503.70	10	50.37			
Lack of Fit	306.23	6	51.04	1.03	0.5110	not significant
Pure Error	197.47	4	49.37			
Cor Total	4644.91	12				

These values conclude that two factors (A-Time and B-Volume) were significant and had significant positive and negative effects on glucose content (**Table S2**). Hence, the experimental values obtained from the central composite design (CCD) were regressed using a linear equation, and the regression equation, expressed in terms of the actual factors, is shown below. The graph of observed values vs. expected values is shown in **Figure S6.**

$$\text{Glucose content} = +86.99585 - 5.53127\ (\text{Time}) + 0.131558\ (\text{Volume}) \quad \textbf{(2)}$$

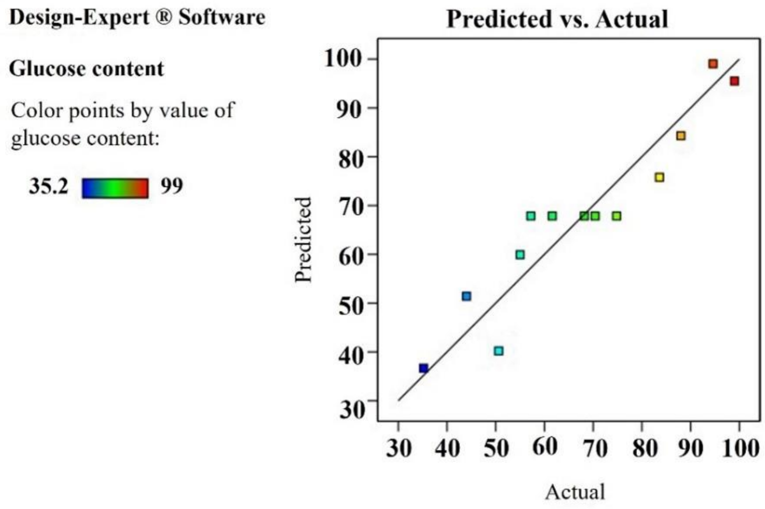

Figure S6. Correlation between the actual and predicted glucose content.

2.2.1. Effects of Variables on Glucose Content

The individual effects of time and volume showed a negative and positive impact on glucose content, respectively, and a one-factor plot for these two factors was generated (**Figure S7**). Based on this figure, it showed that increasing the time interval for the replacement of fresh culture medium from 5 to 13.5 hours had a negative influence on glucose content.

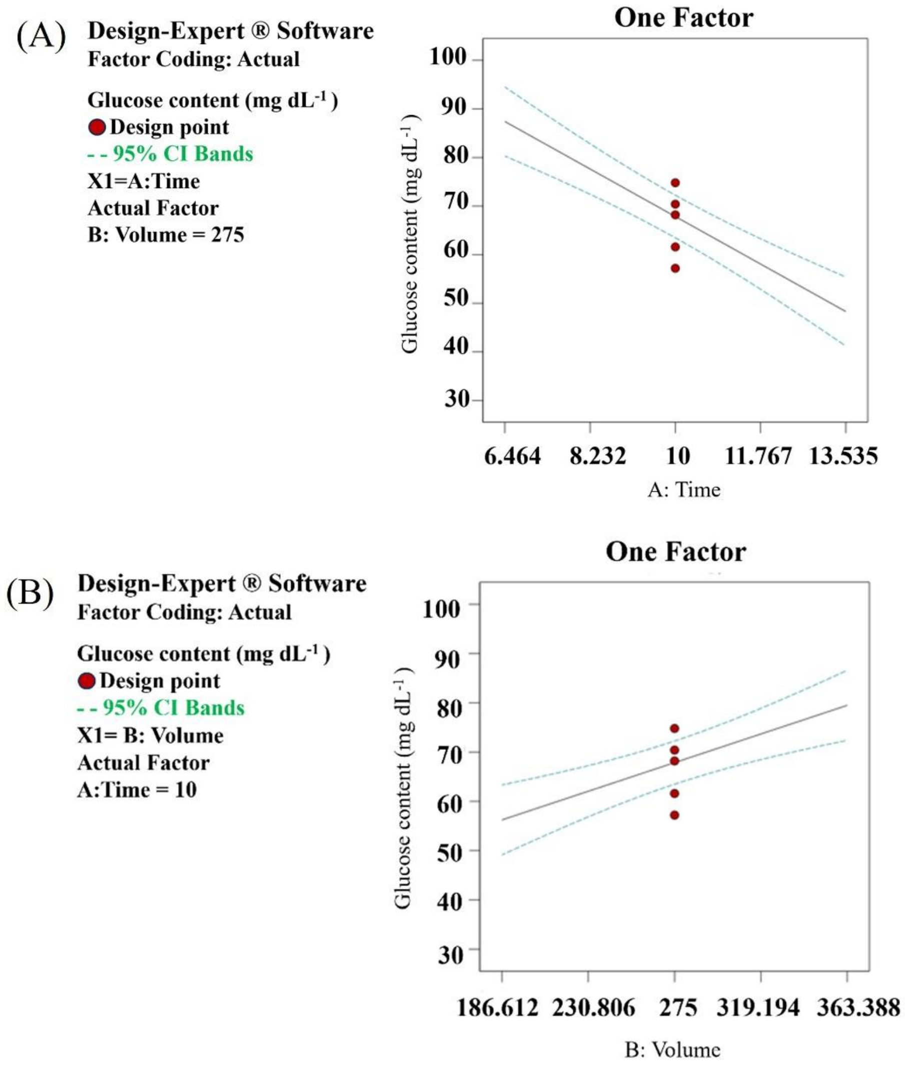

Figure S7. One factor plot of Time (A) (Up) and Volume (B) (Down) on Glucose content.

2.3. Optimization of Exosome Content

The amount of exosome content varied widely, depending on the parameters and concentration of the studied factors, as shown in Table 1 in the method section. The maximum exosome content was in standard order 2 (178×10^3 exosomes μL^{-1}) and the lowest was in standard order 5 (27×10^3 exosomes μL^{-1}), with a maximum-to-minimum ratio of 6.59. A ratio greater than 10 usually implies that a power transformation is required to increase the normality of the dataset. However, since the normal plot of residuals (**Figure S8**) confirmed that the dataset follows a normal distribution, the optimization process was carried out without any power or further transformation.

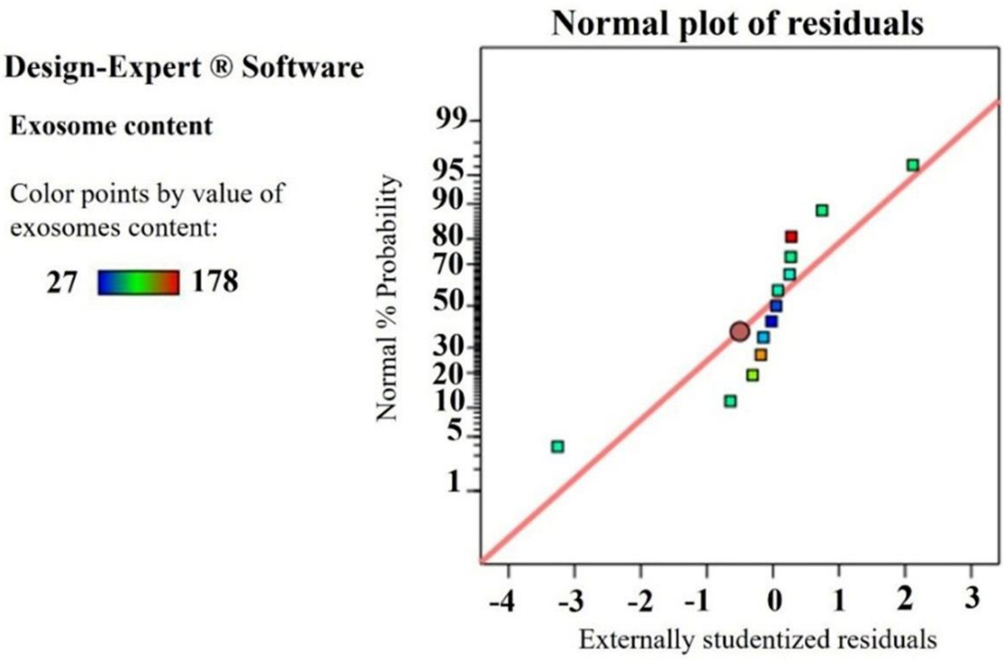

Figure S8. Normal plot of residual of exosome content data distribution.

The sequential model sum of squares, lack of fit test, and R^2 value showed that a quadratic equation was the fittest model for regression of the experimental data. ANOVA for the quadratic model is shown in **Table S3**. These values showed that all factors and their interactions had a significant effect on exosome content. The experimental values obtained from the central composite design (CCD) were regressed using a quadratic polynomial equation, and the regression equation, expressed in terms of the actual factors, is shown below. The graph of observed values vs. expected values is shown in **Figure S9**.

$$\text{Exosomes content} = +81.40 +34.10\,(\text{Time}) -35.73\,(\text{Volume}) -17.25\,(\text{Time} \times \text{Volume}) -3.08\,(\text{Time})^2 +12.17\,(\text{Volume})^2 \quad \textbf{(3)}$$

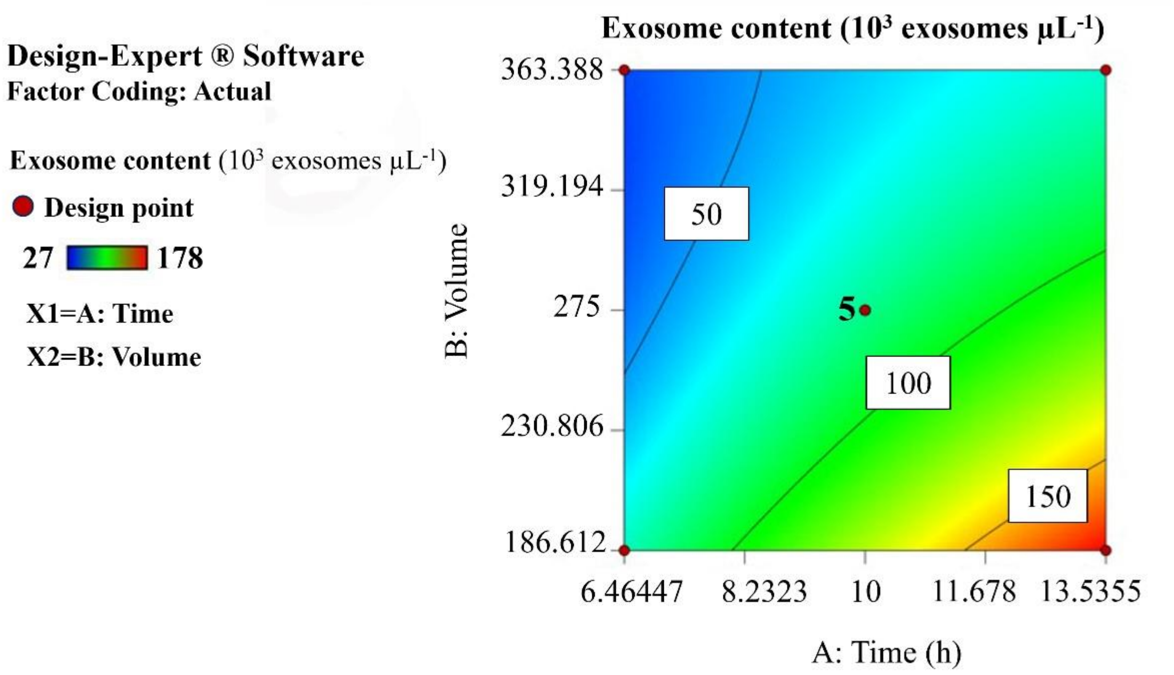

Figure S9. Correlation between the actual and predicted exosome content.

Table S3. ANOVA for exosome content

Source	Sum of Squares	df	Mean Square	F-value	*P*-values	
Model	21888.11	5	4377.62	808.66	< 0.0001	significant
A-Time	9300.06	1	9300.06	1717.95	< 0.0001	
B-Volume	10212.75	1	10212.75	1886.55	< 0.0001	
AB	1190.25	1	1190.25	219.87	< 0.0001	
A^2	65.78	1	65.78	12.15	0.0102	
B^2	1031.17	1	1031.17	190.48	< 0.0001	
Residual	37.89	7	5.41			
Lack of Fit	0.6942	3	0.2314	0.0249	0.9939	not significant
Pure Error	37.20	4	9.30			
Cor Total	21926.00	12				

2.3.1. Effects of the Variables on Exosome Content

As mentioned earlier, all interactions had a significant impact on exosome content (Table S3). The maximum exosome content was obtained when the time interval for the replacement of fresh culture medium (A) was close to the maximum time and the volume of replaced culture medium (B) was close to the minimum volume of replacement, and vice versa, as shown in the contour plot (**Figure S10**).

This is consistent with our expectation that exosome content will increase by lowering the feed flow rate to a level where the medium would no longer be capable of nourishing the cells. Moreover, the results showed that a time interval for the replacement of fresh culture medium of more than 13.5 hours has a negative effect on exosome content, as shown in Figures 3a and 3b, as well as in standard order 7, where only 156×10^3 exosomes μL^{-1} were concentrated when the volume of replaced culture medium was 150 μL, compared to 178×10^3 exosomes μL^{-1} in standard order 2 when the volume of replaced culture medium was 186.6 μL (See Table 1 in the method section). This is likely due to the inhibition of cell proliferation caused by a lack of nutrients and oxygen, which consequently decreased exosome secretion.

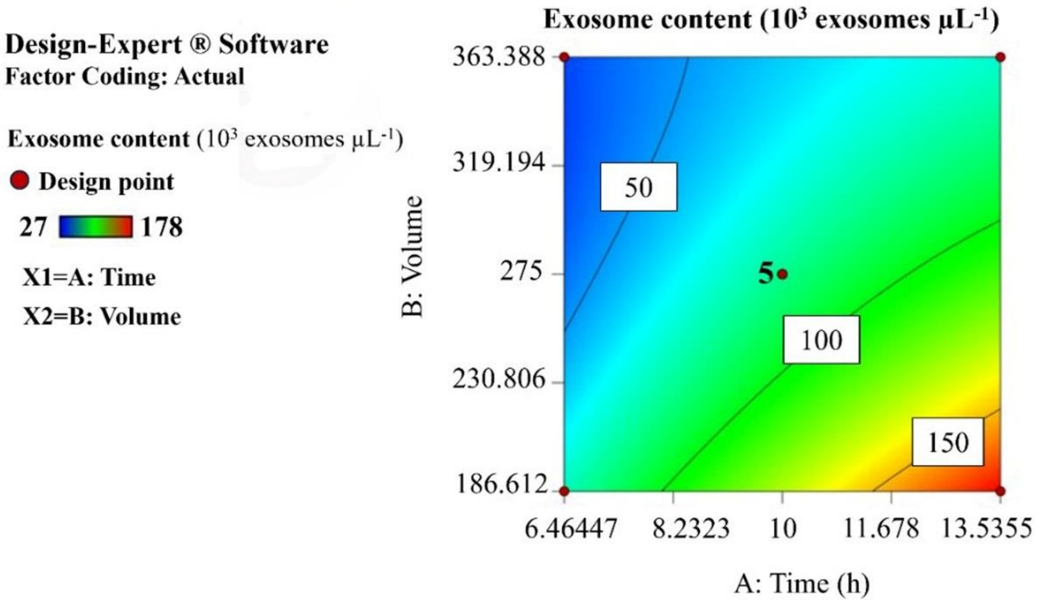

Figure S10. Contour plot for exosome content.

2.4. Optimization Using Desirability Approach in RSM

In RSM-based optimization, analytical and graphical methods are used to determine the optimum process parameters through a desirability approach. The optimized solution can be arrived at based on the chosen criteria and importance level. Based on the regression analysis of the model equation for exosome content, cell proliferation, and glucose content, the optimum levels of the variables were estimated. Various desirability values for all process parameter levels are depicted in **Figure S11**. The optimum conditions for the time interval for the replacement of fresh culture medium were 12 hours and 150 μL for the volume of replaced culture medium. The predicted optimal cell proliferation, exosome content, and glucose content

were 2902 cells mm^{-2}, 189×10^3 exosomes μL^{-1}, and 40 mg dL^{-1}, respectively, according to the model equation (Eqs. 1, 2, and 3) and **Table S4**. **Figure S12** shows the desirability plot, indicating the optimized solution for each process parameter.

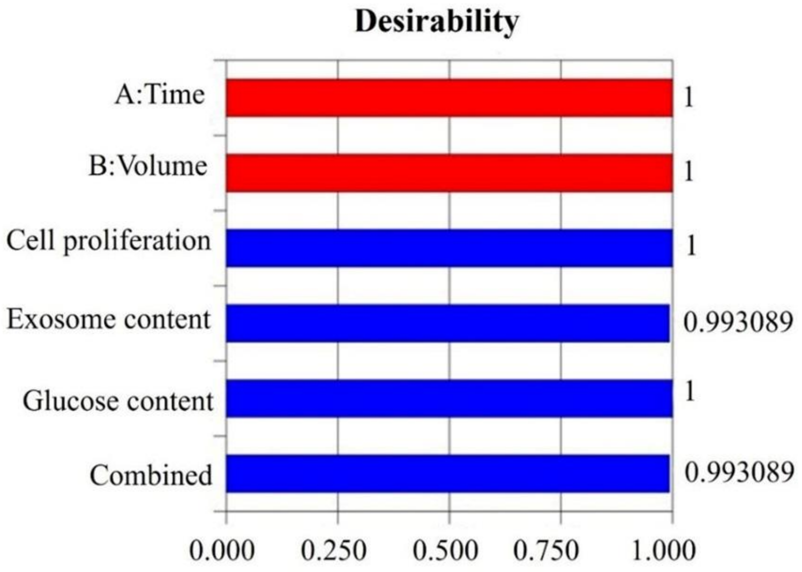

Figure S11. Desirability bar chart for optimum solution.

Table S4. Optimized solutions using RSM.

Number solution	Time	Volume	Cell proliferation	exosomes content	Glucose content	Desirability	
1	12.064	150.000	2902.472	189.378	40.000	0.993	Selected
2	12.184	155.060	2913.472	186.656	40.000	0.963	
3	12.250	157.832	2919.498	185.168	40.000	0.946	
4	11.586	150.000	2934.838	181.901	42.643	0.910	
5	12.476	167.324	2940.134	180.082	40.000	0.890	
6	11.461	150.000	2943.316	179.924	43.336	0.888	

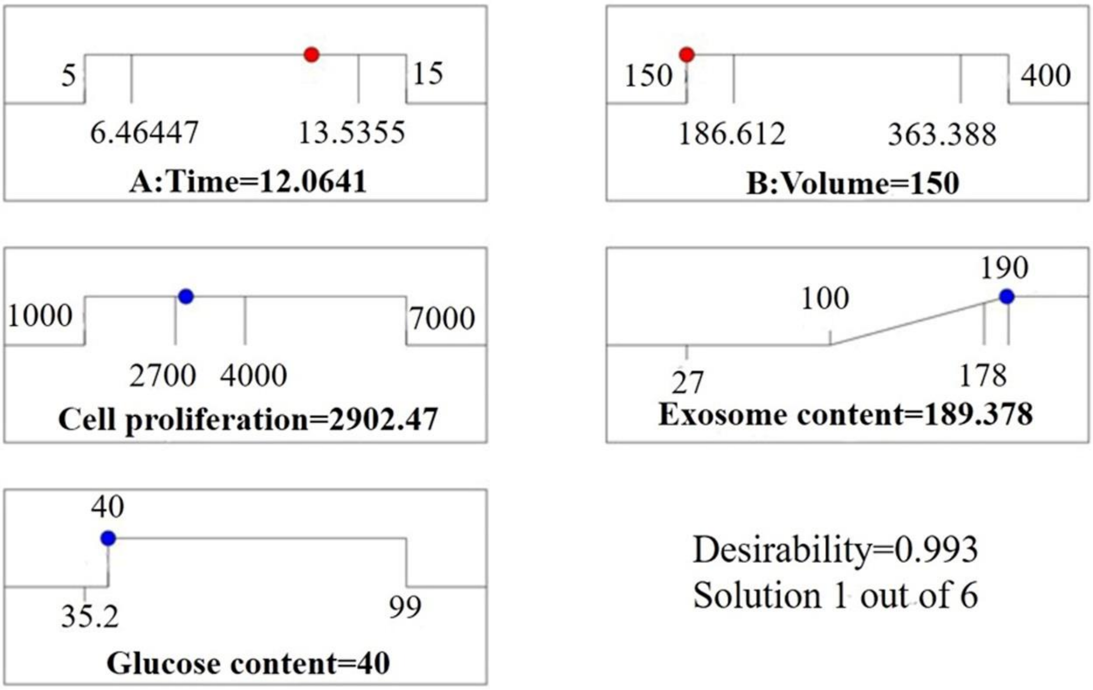

Figure S12. Desirability ramp (graphical representation of numerical optimization results).

2.5. Model Validation

Further experiments were carried out to validate the predicted cell proliferation, exosome content, and glucose content under the estimated optimum conditions generated by the software. The data for cell proliferation, exosome content, and glucose content for each response before and after the optimization are shown in **Table S5**. The exosome content obtained from optimized conditions was 189×10^3 exosomes μL^{-1}, which is comparable to the predicted values and significantly higher than the original medium before the optimization (**Table S5**). Therefore, this experiment validated the model, and the RSM-CCD can be used as an optimization tool to enhance exosome content.

Table S5. Comparison of cell proliferation, exosome content, and glucose content prior and after the optimization.

Parameter	Unit	Before optimization	After optimization
Cell proliferation	Cell number mm^{-2}	3800	2902
Exosome content	10^3 exosomes μl^{-1}	27	189
Glucose content	mg dl^{-1}	99	40

2.6. Statistical Analysis

The linear and quadratic models were fitted to the obtained responses, and analysis of variance (ANOVA) was performed. The coefficient of determination (R^2), adjusted R^2, coefficient of variation (CV), lack of fit, etc., were considered for evaluating the model significance. P-values less than 0.05 indicated that the model terms were significant, while values greater than 0.10 indicated that the model terms were not significant.

B. Additional photos

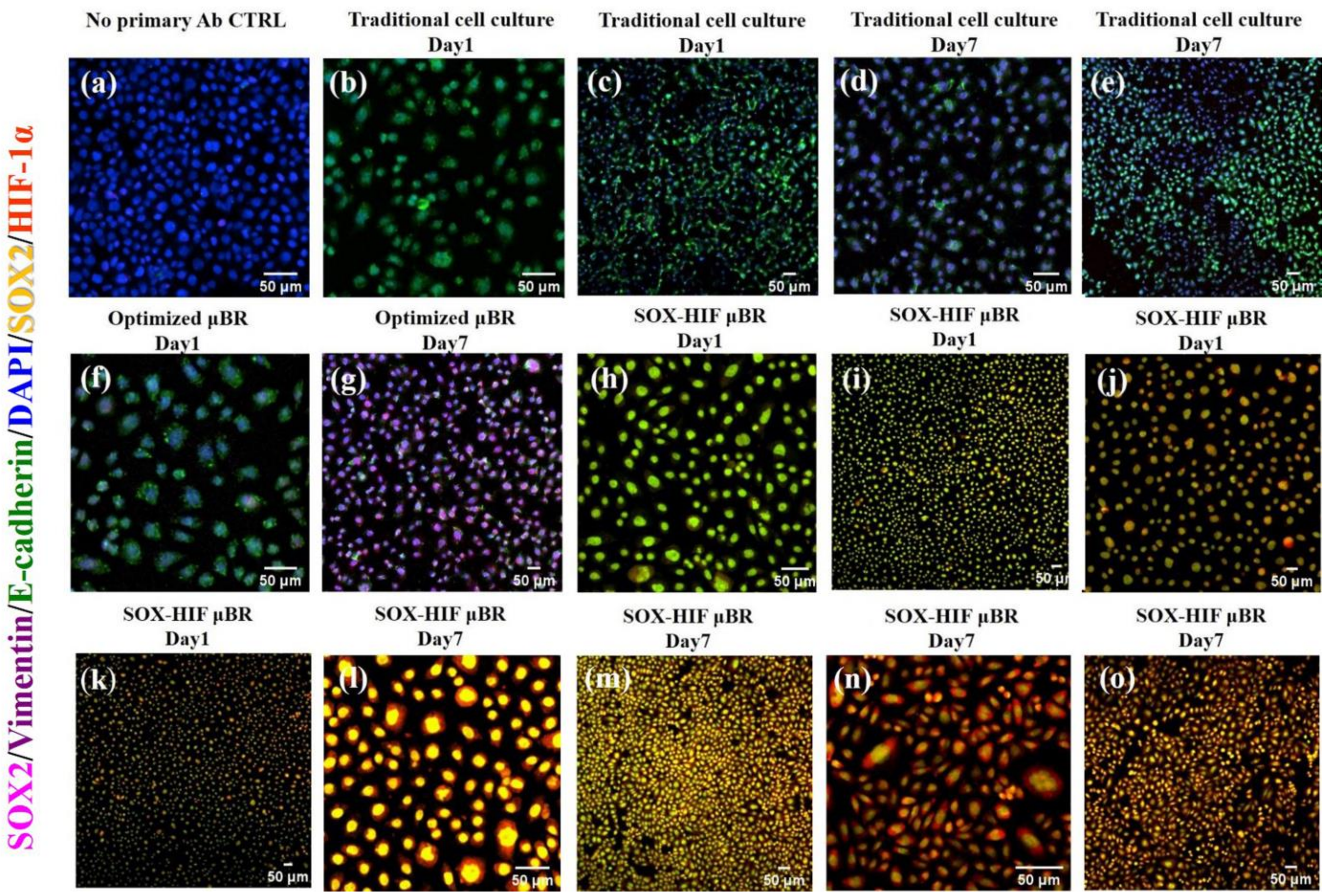

Figure S13. Comparative analysis of EMT and CSC marker proteins in Glioblastoma cell line culture under optimized condition in Microbioreactor vs. Traditional cell culture. (a-g) Immunofluorescence analysis for SOX2 (as a stemness marker) and Vimentin and E/N- Cadherin (as EMT marker) was performed in Glioblastoma cell line culture under optimized condition in µBR vs. Traditional cell culture on days 1 and 7. (h-o) Fluorescent microscopic images of live U87MG cells in optimized µBR by AO staining on days 1 and 7.

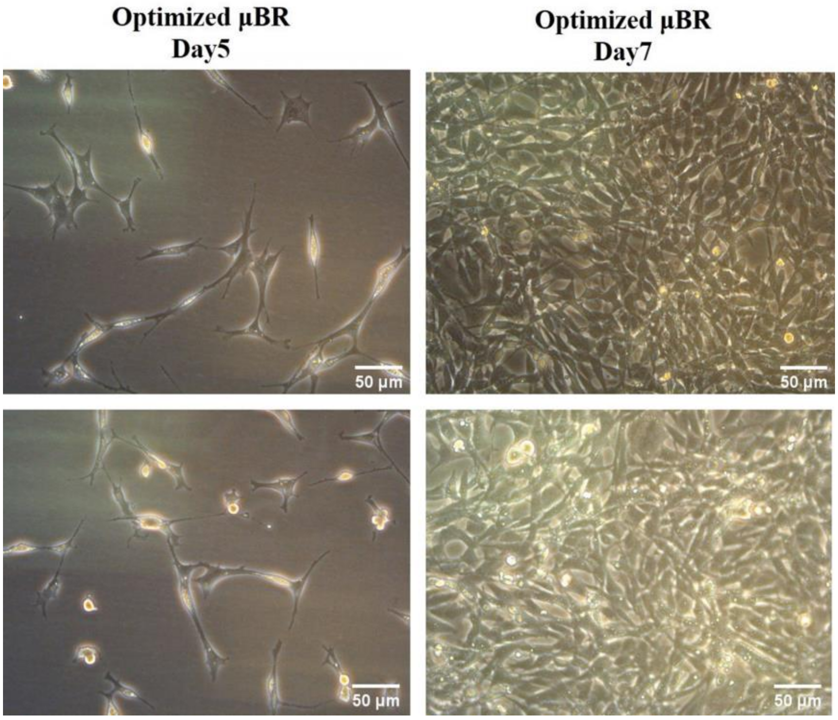

Figure S14. Behavior of Glioblastoma cell line (U87) in optimized μBR.
